# Song type and song type matching are important for joint territorial defense in a duetting songbird

**DOI:** 10.1093/beheco/arab030

**Published:** 2021-06-17

**Authors:** Amie Wheeldon, Paweł Szymański, Adrian Surmacki, Tomasz S Osiejuk

**Affiliations:** 1 Department of Behavioural Ecology, Institute of Environmental Biology, Faculty of Biology, Adam Mickiewicz University, Uniwersytetu Poznańskiego, Poznań, Poland; 2 Department of Avian Biology and Ecology, Institute of Environmental Biology, Faculty of Biology, Adam Mickiewicz University, Uniwersytetu Poznańskiego, Poznań, Poland

**Keywords:** duetting, song matching, song sharing, song type function, song type repertoire, territorial defense

## Abstract

Birds have a diverse acoustic communication system, with species-specific repertoires facilitating more complex behaviors in terms of both within- and between-pair communications. Certain song types are produced for specific functions, such as aggressive encounters. In addition, song matching behaviors, whereby neighboring individuals match song types, can be used in aggressive interactions as a sophisticated acoustic behavior. In this study, we examined the functions of song types, in a duet context, of male yellow-breasted boubous (*Laniarius atroflavus*), an Afromontane bush-shrike with a vocal sexual dimorphism. We aimed at assessing whether, structurally, certain song types elicited a heightened reaction than others and also whether song matching affected response behavior. A dual speaker playback procedure was performed for 18 pairs of boubous, each pair being exposed to duets with three different male song types. We found differences in response toward the different duet types but these differences resulted from the amount at which males matched different song types. Pairs responded stronger when a focal male matched the playback type, and matching was significantly more often found in cases where the rarest type of male song was used. We found no sex differences in terms of response strength to playback type. Our results indicate a two-level way of coding aggression toward intruding pairs. The yellow-breasted boubous utilize their repertoires, linking matching with structure in order to show aggression in terms of territory defense and sexual conflict. This study also confirms joint territorial defense as a main function of duets in this species.

## Teaser

The diverse song repertoires of birds, in terms of size and organization, facilitate both within- and between-pair communication. Using playback experiments, we showed that males and females of a duetting Afromontane bush-shrike jointly defend territories. The small and shared repertoire of male songs is important for conflict resolution. Our results indicate a two-level way of coding aggression toward territory intruders. Both, song type used and matching a male intruders song type, shape the response strength.

## INTRODUCTION

Communication underpins virtually any animal behavior (Bradbury and Vehrencamp 2011). Signals convey crucial information in sexual selection, affecting both female choice and male–male interactions (Berglund et al. 1996). Information encoded in signals enables contact maintenance (Mumm et al. 2014), individual recognition (Tibbetts and Dale 2007), alarming (Macedonia and Evans 1993), or begging for food (Godfray 1991) to mention just a few important contexts. Some communication systems are highly specialized due to the specific functions (e.g., mammal–pollination systems, Johnson et al. 2011) or unique mode or medium (e.g., flashing of bioluminescent lampyrid beetles or the creation of water ripples by water striders; Bailey 2003). While others, in evolutionary terms, seem to have exploded with overwhelming diversity of forms and functions. Bird song is one such signal type, the most spectacularly diverse acoustic communication system in animals. Although its variability and functions have been studied for many years and are largely well known, a number of aspects of singing remain understudied and are still not well recognized, for example, female song and the role of small repertoires (Slater 2003; Catchpole and Slater 2008). This is particularly important because some of these less well-studied aspects of song seem to be fundamental to understanding the evolution of this behavior (see recent reports on the ancestral nature of female song and its functions in Tobias et al. 2011; Odom et al. 2014; Riebel et al. 2019). One of the multiple dimensions of song variation is repertoire. Put simply, “having a repertoire” means the song of an individual consists of a number of acoustically different units usually referred to as syllables or song types (Snyder and Creanza 2019). In temperate birds, which are disproportionally more studied than their tropical counterparts, it is mainly males that sing and—especially in species with larger repertoires—repertoire size seems to be correlated with various aspects of the sender’s quality with larger or more complex repertoires being preferred by females (Hasselquist et al. 1996; Reid et al. 2004; Hesler et al. 2012). On the other hand, a high proportion of bird species have a small repertoire, starting with a single song type up to a dozen or so, and individuals within a specific population do not usually have a large difference in the repertoire size they possess (MacDougall-Shackleton 1997; Catchpole and Slater 2008). It seems, therefore, that not only the size of the repertoire but also its specific composition or other features may play an important role in intersexual selection (Gil and Gahr 2002; Slater 2003).

An important aspect of song repertoire evolution is the way in which repertoire units are used during interactions with other individuals (Beecher et al. 2000). One of the most interesting phenomena in this context is the sharing of repertoires between individuals within a population and the consequent use of song matching. Song matching is a form of vocal matching that can be defined as an interactive process in which an individual intentionally produces an identical (or very similar) signal as any other individual to whom the signal is addressed (King and McGregor 2016). Such a system enables the transmission of the signal toward a particular receiver as well as providing information about the motivation of the signaler (Searcy and Beecher 2009). It is not an avian-specific behavior and has also been found in distant animal groups, for example, cetaceans (King et al. 2013). It is assumed that the song matching systems have a conventional character (Vehrencamp 2001; Akçay et al. 2013). In other words, producing a particular type of signal does not involve any particular cost in comparison to the other, which would be due, for example, to the fact that it is more energetically costly to produce. The evolutionary stability of such a communication strategy is due to the costs associated with the receiver’s response. For example, if matching the song type of an intruder (Akçay et al. 2013) or any other conventionally relevant acoustic signal (e.g., Ręk and Osiejuk 2010) provides information about heightened aggression, the potential cost for the sender is getting involved in a physical fight. Hence, signaling readiness to fight is only worthwhile if the sender honestly signals its physical strength or motivation to defend resources (Guilford and Dawkins 1995). Such systems have indeed been shown experimentally, but we still do not know how widespread they are and what factors promoted their evolution. One of the reasons for that is the scarcity of research conducted on different model species as the sharing and matching of repertoires can be substantially different between species and even between different populations of the same species (Searcy et al. 2019). Even in the best studied model, the song sparrow *Melospiza melodia*, different research presents dissimilar results, most likely because populations with a different ecology differ in the way they communicate. In western populations of the song sparrow, the early matching of a song type predicted subsequent attack (Akçay et al. 2013), whereas in eastern populations matching did not result in a stronger response (Searcy et al. 2013). Moreover, song sharing and matching may also be a result of different processes. For example, different song units may have different functions resulting from their structure (Byers 2017) or they may be stochastic results of learning and dispersal processes without any obvious effect on male–male interactions (Podos and Warren 2007). Interestingly, species with both medium- and large-sized repertoires may sing a given song type only in specific behavioral contexts. For instance, male chestnut-sided warblers *Setophaga pensylvanica* are much more likely to use their rare song types in extremely aggressive territorial interactions than during spontaneous singing (Byers 2017). In addition, the different song categories of this warbler species may also encode information on male’s location, status, and behavioral tendencies (Byers 1996). What’s more, various song categories and/or singing modes are suggested to be specifically directed to either male or female receivers (Spector 1992), but such a distinction is not always easy and meaningful (Beebee 2004). The mechanisms as to how these rare song types act as an aggressive signal are still poorly understood. Similar issues have been investigated in male-only singing species and have shown that different song categories may encode different information about male status, location, and behavioral tendencies (Byers 1996). The different song categories (or modes of singing) are sometimes suggested to be specialized for male or female receivers (Spector 1992), but such a distinction is not always easy and meaningful (Beebee 2004).

In terms of singing behavior, tropical birds are relatively understudied compared to temperate zone birds. In many tropical species not only males, but also females, sing and both sexes perform vocal displays in duets. Therefore, in the context of duetting hypotheses that different song types convey different messages, to the best of our knowledge, have not be really been addressed. More attention has been paid on decoding the answering rules and describing how females and males choose song types from their repertoires to build a common duet (review in Hall 2009). Duetting species are more common in tropical regions. This behavior has evolved several times in a range of different bird species from phylogenetically distant groups (Odom et al. 2014) but occurs most often in sedentary and group living species (Soma and Brumm 2020). The role of duetting in bird communication seems to be much more complicated in comparison to solo singing and is still a relatively understudied area demanding intense research (Hall 2004, 2009). It is important to point out that duetting birds also have repertoires in their song. Males and females of duetting species may differ in the size and level of sharing of song repertoires resulting in radically different strategies of repertoire use. Knowledge about specific repertoire use (e.g., song type matching) in duetting species is very poor; so far only a few studies have addressed this topic directly. These studies indicate that song matching in duetting species has a variety of functions as it seems to serve for both territory defense and within-pair communication in some cases (e.g., Marshall-Ball and Slater 2004; Rogers 2005; Rogers et al. 2006; Moser-Purdy et al. 2019).

**Figure 1:**
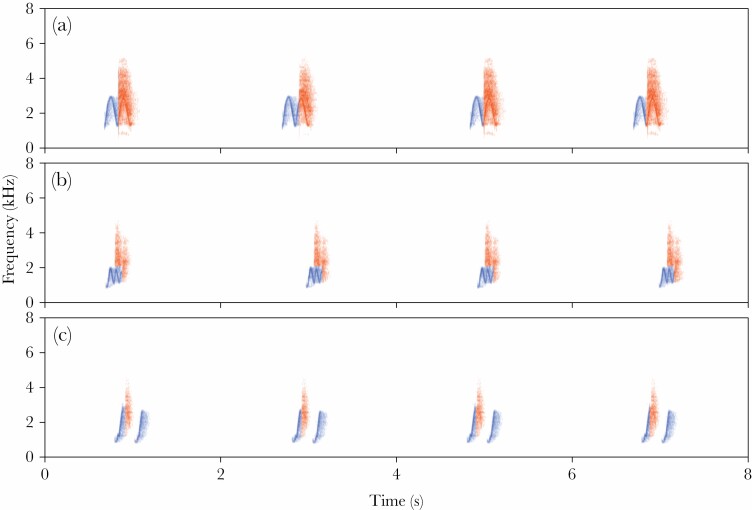
Spectrograms of the yellow-breasted boubou duet types. The blue color indicates male and the red color indicates female song phrases. (a) A duet composed of a male High hwee-oo phrase and a female Chock phrase (abbreviated as HC); (b) a duet composed of a male Low hwee-oo phrase and a female Chock phrase (abbreviated as LC); and (c) a duet composed of a male Hwee-hwee phrase and a female Chock phrase (abbreviated as WC). Spectrograms illustrate typical duets produced by birds; those used for playback were standardized by the use of a single Chock with the same delay as a male phrase. The characteristic fixed intervals between phrases produced by pairs is clearly visible.

In this study, we investigated the role of using specific song types and song type matching in a duetting songbird, the yellow-breasted boubou (*Laniarius atroflavus*). The yellow-breasted boubou is a bush-shrike endemic to Western Africa with a unique type of repertoire sharing between males. In this species, both males and females sing solo songs and join partner’s vocalization to form duets. They have very small and sex-specific vocal repertoires, with males producing whistles and females atonal and harsh notes (Riegert et al. 2004; Fry 2020; Wheeldon et al. 2020; see the Supplementary Video). Males are much more vocal than females and most of the overall singing activity is due to the male solos and duets initiated and led by males (Wheeldon et al. 2020, 2021; Szymański et al. 2021). The majority of duets (85%) consist of a male phrase, followed by a female phrase, partially overlapping her mate. Such a male–female phrase is regularly repeated several times in a bout, and such duets were used for playback in the present study (Wheeldon et al. 2020). All males in the studied population share three whistle song phrases, which are used for solos and duets and are delivered with eventual variety (Figure 1). Males very rarely (<1% of cases) switch to a different song type within a bout, both when soloing and duetting (Wheeldon et al. 2020). An interesting feature of this full sharing of the song repertoire pattern is that it is interindividually consistent. All males share the same song type phrases and all use them with similar proportions. The proportions of how often the three song phrase types (i.e., High whee-oo—hereafter H, Low whee-oo—hereafter L, and Hwee-Hwee—hereafter W; Figure 1) were produced by males as solos was 4:2:1 and 5:4:1 when they were answered by a female to form a duet (Wheeldon et al. 2020). Song bouts of male solos and duets are loud (90–103 dB SPL at 1 m) and on average relatively short (24–30 s), but in some situations, they can last for several minutes. Males usually sing a single song type within a bout but they use all three types of their sex-specific signals during the whole day activity and the whole year activity without any clear time pattern (Wheeldon et al. 2020; Szymański et al. 2021). There are only a few exceptions to this. For example, males usually start the dawn chorus with a solo vocalization and they more often than expected by chance start it with the most rarely used song phrase W (~64% vs. expected 15%). However, this song type is not used exclusively during the dawn chorus (Wheeldon et al. 2020; Szymański et al. 2021). Although male song phrases are shared by all individuals within the local population, they have an individually specific structure facilitating individual recognition (Linhart et al. 2019; Wheeldon et al. 2020). The combination of complete song type sharing and individuality of songs suggests that singing may play an important role in male–male interactions as it allows to recognize and address specific individuals by song type matching.

We designed an interactive playback experiment allowing us to test a few basic hypotheses concerning the fully shared song pattern and functions of male song types. As male solos, male-initiated and male-led duets are the most common and the loudest signals produced by the yellow-breasted boubous, we assume that this singing behavior is a signal primarily directed to neighbors or unknown rivals, but this does not rule out simultaneous intrapair communication. If the various song types in a male’s repertoire carry different information about their aggressive motivation for territorial defense (H1), we expect that the response of territory owners to playback of each song type of an unknown intruder will differ. Alternatively, the information conveyed by male singing may not be encoded in the type of song but rather in the interaction between signals of territory owners and intruders (H2: song matching hypothesis). If this is true, one should expect that the differences in response to playback would depend on whether or not a song matches a particular type. For example, a territory owner may respond in a stronger way to the playback that matches the song type they used before the playback or may respond in a stronger way if the responding bird switches to match the playback. These two hypotheses are not mutually exclusive because song matching may act together with basic information about signaler aggressiveness contained in song types.

## METHODS

### Study area and population

This study was conducted in the Bamenda Highlands in Cameroon (6°5’ to 6°8’ N and 10°17’ to 10°20 E). The study area is covered by montane forest patches, shrubby corridors, grasslands, and rocky outcrops at elevations from 1900 to 2400 m above sea level. The yellow-breasted boubou is an insectivorous bush-shrike, endemic to the montane forests of SE Nigeria and W Cameroon. In the study area it is one of the most common species (Reif et al. 2006). Males and females are sexually monochromatic and inhabit dense natural forest undergrowth and human changed habitats, such as the edge of clearings or secondary scrubs (Stuart 1986; Borrow and Demey 2001; Riegert et al. 2004; Fry 2020). The study was conducted at the beginning of the dry season in 2016 (November–December), coinciding with the beginning of the breeding season of the study species (Serle 1981; Tye 1992; Sedláček et al. 2007). Prior to experiments, territorial pairs were intensively observed in order to gain good information about their territories and song post locations. Practices included mist-netting and color ringing as well as following and recording birds whilst taking notes about counter-singing pairs and individuals.

### Sound samples and playback preparation

Songs used in the experiment were locally specific, but the duets played back to particular focal pairs originated from nonneighboring birds recorded at least 1 km away. The core territory where yellow-breasted boubous usually sing rarely exceed 50–80 m (Osinubi 2012; Wheeldon et al. 2021) and birds seem to fill the entire available space (own unpublished data). Hence, between focal pairs and the pair from which song samples came, there were at least several territories of other birds. Birds were recorded at a 48-kHz sampling frequency and 16-bit resolution with a Marantz PMD670 recorder (Marantz Professional, Kanagawa, Japan) or Olympus LS-10, LS-11 or LS-12 (Olympus, Japan) coupled with a Sennheiser ME67 shotgun microphone (Sennheiser, Wedemark, Germany). We selected songs with a high signal-to-noise ratio assessed visually based on sound spectrogram (typically over 40 dB), classified both as solos and duets. All recorded songs were filtered (high-pass, 0.5 kHz; low-pass 16.0 kHz) before the preparation of playback recordings.

Each playback stimulus was created from a single male and female song phrase to compose a natural sounding duet. Each duet was prepared from samples belonging to different individuals to avoid pseudo-replication and to make sure that all tested pairs were responding to unknown individuals each time. During each treatment, we played duets with the same natural rate of about one duet every 2 s. We created duets with the three types of male whistle song phrases: H, L, W. The female part of the duets was always built with the single “Chock” song type (hereafter C), which is commonly used by females in duets when responding to any of the three male song types (Figure 1). All playbacks of duets (hereafter HC, LC, and WC) reflected the most common situation, where a male initiates and leads a duet, while a female follows the partner’s phrases with a short delay and overlaps each of his phrases with a single female phrase. The volume of each playback was set to 90 dB SPL at a 1 m distance from the speaker, measured with a CHY 650 digital sound level meter (CHY Firemate Co., Ningbo, China). Digital editing, construction, and analysis of the playback files was conducted with Raven Pro 1.4 (Cornell Lab of Ornithology, Ithaca, NY).

### Playback procedure

Playback experiments were performed between November 13 and December 1, 2016 during the first 4 h of the morning (6:30–10:30 AM local time). Altogether, we tested 18 territorial pairs three times, with the three types of duet (HC, LC, and WC) presented in a counterbalanced design. The three treatments for a particular pair were done on different days and were separated by 24–48 h. Experiments were only conducted when both the male and female were present before the playback. In each trial with a given pair, loudspeakers were placed in a slightly different way to avoid habituation of focal birds but always within a few meters from a central point of a breeding territory.

**Figure 2:**
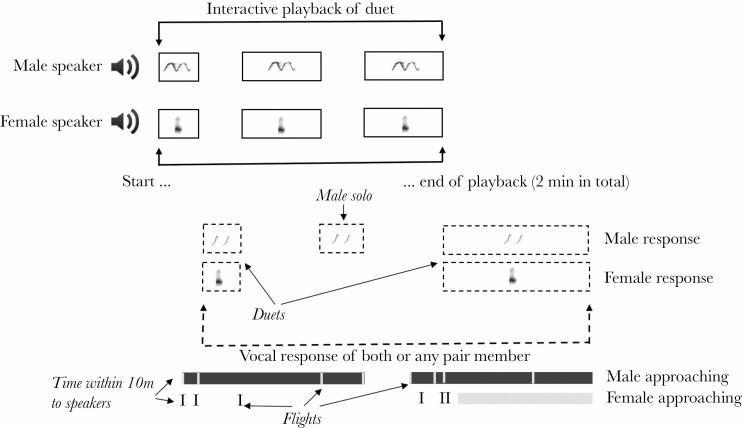
Illustration of the course of the experiment. Duets were played back from two speakers for 2 min in total but were paused as soon as the focal pair (or any pair member) started their vocal response and started again when birds stopped responding vocally. After the end of the playback, the response of birds was recorded for the next 2 min. Simultaneously to this recording, the physical response movements and distance to speakers were noted.

The male and female phrases were played back as separate channels of a WAV file with an Apple iPod Touch player (model A1574, Apple Inc.) and from two UE Boom speakers (Logitech, Lausanne, Switzerland) placed on branches ca. 2 m above the ground and separated by 9.8 ± 0.27 m (95% CI: 9.2–10.3 m) on average. Such a positioning of the speakers reflected a natural situation of focal pair member positioning and enabled us to quantify the response of focal birds and whether a certain behavior was specifically directed toward the simulated male or female (speaker). We only began the experiment procedure when the tested pair was singing within their territory, which sometimes led to a wait of up to 60 min. When singing started, birds were observed and recorded until they stopped spontaneous vocalization, and then we started the playback of 2 min of a duet, simulating the intrusion of a stranger pair. However, when we heard the focal pair had started responding vocally, we immediately stopped the playback and waited for the pair or pair member to also stop singing. When the focal birds stopped responding vocally, we started playback again. This stopping and starting of the playback lasted until we played the full 2 min of the prepared playback. Hence, the playback period of the experiment lasted at least 2 min plus the time when focal birds were responding vocally. After this phase, we observed the focal pair for 2 min of the postplayback period (Figure 2). The interactive playback approach we used was designed to match the behavior of the study species. First, it reflected the way birds sing naturally with clear ends of song bouts and longer movements between singing sessions. Second, this approach allowed the avoidance of overlapping focal birds’ song by the playback, which might have affected their response in an uncontrolled way, especially as yellow-breasted boubous are very loud, often singing with amplitude over 90 dB SPL. Duets are formed by male phrases repeated with very stable intervals within a bout with a female usually adding a phrase just after the start of a male phrase (as presented on Figure 1). Hence, our approach was the only way to keep the natural rate of duetting without overlapping responding birds or adding uncontrolled delays between subsequent phrases from the focal pair and playback, and presenting all tested pairs with the same duration of playback. Third, birds that were responding in a strong way usually started the response very soon after the playback started and the overall response during the whole experiment was strongly correlated with this initial response. On the other hand, if a very short playback had been used, this would increase the chance of no vocal response from less motivated birds. A similar design was used in a previous study on a loud rail species where simulating an interaction without overlapping the tested birds’ calls was crucial (Ręk and Osiejuk 2010, 2011).

All vocal responses of the tested birds were recorded using an Olympus Ls-11 recorder, coupled with a Sennheiser ME67 shotgun microphone (Sennheiser, Wedemark, Germany) by one of the observers positioned ca. 20 m from the speakers. The second observer was usually located on the other side of the speakers in a place convenient for the observation of the whole experimental scene. The birds’ behavioral responses were recorded by dictating observations into the lavaliere microphone attached close to the observer’s mouth, which made it possible to use binoculars when necessary. We started recording well before the start of the playback in order to assess the situation and the position of the focal birds. Recordings of vocalizations and physical behavior were later synchronized based on the “beep” sound included in playback at the end of the postplayback period. All distances between the speakers and for the responding birds were measured with a Leica DISTO D510 laser range finder. The closest distances to a speaker and between speakers etc. were measured just after the end of the experiment.

We tried to record many aspects of the focal birds’ behavior but for the aim of the analysis we limited it to those variables that we were able to collect with sufficient and repeatable certainty among all experiments. In the case of the vocal response, we used number of phrases sung by males and females in duets and separately the number of phrases sung by males and females in solos. As yellow-breasted boubous are loud, there was no problem with assigning every single phrase to a particular sex and type. We also noted if responding males matched the same type of song as used for the playback. We assigned the response as matching if the male started the response with the same song type as used for the playback (in one experiment, the male switched to match the playback after singing three phrases of the previously sung type). The physical behavioral responses of males and females were measured by the time spent within 10 m to speaker (s), latency to approach the speaker (s), closest approach to the speaker (m), and number of flights.

### Ethical note

To our knowledge, the individuals tested in the experiment reflected the population in a representative way with no potential biases resulting from social background, self-selection, habituation, or other factors as indicated in the STRANGE framework (Webster and Rutz 2020). All work adheres to the Association for the Study of Animal Behaviour (ASAB)/Animal Behavior Society (ABS) Guidelines for the Use of Animals in Research. Our study was approved by the Local Ethical Committee for Scientific Experiments on Animals, University of Life Sciences, Poznań (permission no. 16/2015) and the Polish Laboratory Animal Science Association (certificate no. 1952/2015 to T.S.O.) conforming to Directive 2010/63/EU.

### Statistical analysis

We present basic descriptive statistics, starting with summaries of how many pairs responded to different treatments vocally and by approaching the speakers. Such binary response variables as vocal response (duet and solo), approaching or close approaching (<10 m), as well as matching the playback type by focal male, were useful for the general description of bird responsiveness and tested with χ ^2^ or χ ^2^ with Yates’ correction tests. For the quantitative analyses, we analyzed two vocal response measures reflecting the number of phrases sung in duets and solos and the four aspects of approaching (latency, closest distance, time in 10 m to speakers, and flights). Means are presented ± standard error throughout and males and females are always treated separately. We analyzed quantitative data using generalized linear mixed models (GLMM) to comply with nonnormal data (Bolker et al. 2008). Our response variables were log-transformed measures of vocal response (dues and solos) and approaching (latency, closest distance, time in 10 m to speakers, and flights). We included the following main factors in our models: 1) playback treatment (three levels: HC, LC, and WC duets); 2) playback order (three levels: first, second, or third); 3) sex of responding bird (two levels: male or female). We used sex as a factor because duetting is not the only type of vocal activity used by the yellow-breasted boubou, and sexes may potentially respond differently to playback (Wheeldon et al. 2020, 2021). A similar approach was used in an earlier study on different duetting species (Kovach et al. 2014). We used log linear target distribution and included all first-order interaction terms, and we incorporated pair identity as a random effect. To test the effect of treatment on playback type matching, we used a GLMM with a binomial error structure and logit link function. In this analysis, only males’ response variable was tested as only males might match the male part of the playback. Post hoc pairwise comparisons were obtained through the GLMM interface with *P* values corrected for multiple comparisons using the least significance difference (LSD). The models’ parameter choice was based on the diagnosis of response variables distribution, inspecting the QQ plot and the lowest corrected Akaike information criterion criterion, as available in GLMM panel in SPSS 26 (IBM, Chicago, IL) used for these analyses.

The last aspect of bird response we focused on was song type matching. As males in the studied population share all sex-specific song types, they could potentially freely decide if they respond with the same or different song type to playback. One can imagine that if males respond by choosing the song type randomly from the repertoire, the chance of matching the playback would be 33.3% (1 divided by 3 types available). However, assigning a random matching pattern to yellow-breasted boubous seems to be a very superficial reflection of natural behavior. An earlier study showed that the song types H:L:W were not used in equal proportions (Wheeldon et al. 2020). Depending on whether we consider solos or duets, the proportions were 4:2:1 or 5:4:1, respectively. So matching song types in experiments should be compared to such frequencies.

## RESULTS

We found that out of 54 trials with 18 pairs tested, focal birds responded with duet in 36 of the experiments (66.7%); in 13 experiments (24.1%), males produced solos; and in 10 experiments (18.5%), females sang solos. However, in only six experiments, solos were the only vocal response that was produced (four male solos and two experiments for females). Approaching of males was observed in 44 experiments (81.2%) and in 41 experiments (75.9%) for females. Males approached within a 10 m distance to the speakers in 25 experiments (46.3%) and females in 24 experiments (44.4%). We found no statistically significant differences in the distribution of all the above binary responses between treatments as well as between sexes within response category (all *P* > 0.197 for χ ^2^ or Yates’ χ ^2^ tests).

**Figure 3:**
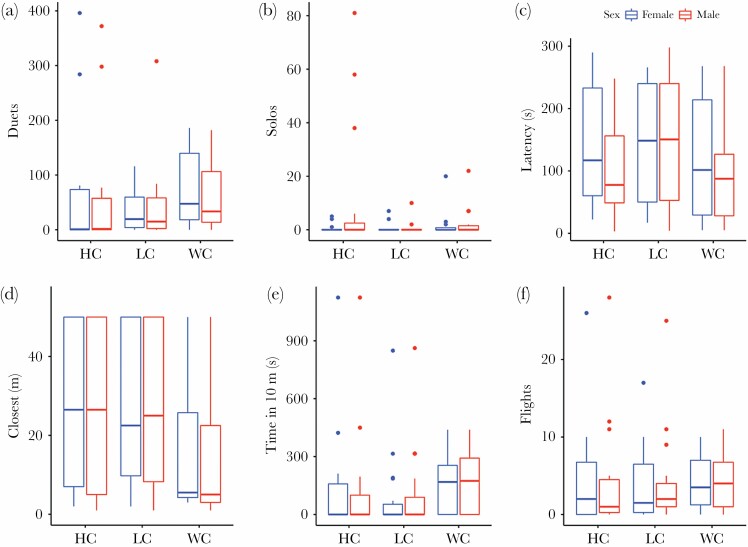
Female and male responses to playback of different duet types (HC, LC, and WC): (a) number of phrases produced in duets, (b) number of phrases produced solo, (c) latency to respond (s), (d) closest distance of approach to speaker (m), (e) time spent in a distance within 10 m to speaker (s), (f) number of flights to speakers. Box plots show the median with a horizontal line, the interquartile range (25th–75th percentile) with boxes, and the values within 1.5 times the interquartile range with whiskers. Dots show values exceeding 1.5 times the interquartile range.

Quantitative measures of response to different playback types are presented in Figure 1 and relevant tests are presented in Tables 1 and 2. Male and female measures of response within a particular response category were highly correlated, and we did not observe significant sexual bias in the response, including in the response to different playback types (Tables 1 and 2). We found a significant effect of the order of experiment (*P* < 0.001) and treatment (*P* = 0.007) on the number of song phrases produced by males and females in duets (Table 1). On average, focal birds produced significantly less phrases in duets in following trials with the same pair, on average in the first: 84.4 ± 16.80, second: 50.7 ± 11.96, and in third: 27.4 ± 7.63. The paired post hoc comparisons revealed significant differences between first:second and first:third experiment (*P* ≤ 0.001) but not between second:third (*P* = 0.476). As for the effect of treatments, we found the strongest duetting response to the WC playback, then to the HC playback (post hoc *P* = 0.002), and finally to the LC playback (post hoc *P* = 0.052; Figure 3a; Table 1). Solo responses to playback were relatively rare (Figure 3b) and when this happened, birds never produced as many phrases as duets. The only significant effect was found for the Treatment × Order interaction (Table 2), which was the result of many more solos (15.5 ± 8.03) produced when birds responded for the first time to the HC treatment (post hoc *P* = 0.009). However, this was only observed in a few experiments and usually males produced less solos (2.7 ± 1.01).

**Table 1:** Factors and interaction terms from the generalized linear mixed models used to analyze vocal responses of the yellow-breasted boubou to playbacks simulating intrusion of a stranger pair singing three different duet types

	Number of song phrases produced in duets			Number of song phrases produced solo		
	*F*	df	*P*	*F*	df	*P*
Treatment	5.30	2,94	**0.007**	1.96	2,94	0.146
Order	9.89	2,94	**0.000**	1.93	2,94	0.151
Sex	0.09	1,94	0.770	2.58	1,94	0.112
Treatment × Order	2.20	4,94	0.075	3.24	4,94	**0.015**
Treatment × Sex	0.03	2,94	0.974	1.74	2,94	0.181
Order × Sex	0.02	2,94	0.981	1.41	2,94	0.249

The values in bold indicates significance at *P* ≤ 0.05 level.

**Table 2:** Factors and interaction terms from the generalized linear mixed models used to analyze four aspects of approaching behavior responses of the yellow-breasted boubou to playbacks simulating intrusion of a stranger pair singing three different duet types

	Latency to approach speaker (s)			Closest approach to the speaker (m)			Time spent within 10 m to speaker			Number of flights		
	*F*	df	*P*	*F*	df	*P*	*F*	df	*P*	*F*	df	*P*
Treatment	2.46	2,94	0.090	4.93	2,94	**0.009**	6.22	2,94	**0.003**	1.58	2,94	0.213
Order	5.93	2,94	**0.004**	4.00	2,94	**0.022**	3.77	2,94	**0.027**	8.08	2,94	**0.001**
Sex	0.98	1,94	0.325	0.34	1,94	0.562	0.15	1,94	0.696	0.01	1,94	0.906
Treatment × Order	1.16	4,94	0.334	0.96	4,94	0.433	1.06	4,94	0.380	3.66	4,94	**0.008**
Treatment × Sex	0.19	2.94	0.824	0.00	2,94	0.997	0.03	2,94	0.971	0.19	2,94	0.830
Order × Sex	0.29	2,94	0.749	0.00	2,94	0.996	0.04	2,94	0.964	0.02	2,94	0.981

The values in bold indicates significance at *P* ≤ 0.05 level.

All approaching variables (Table 2) were significantly affected by the order of experiment. Birds were less likely to approach quickly and closer when they were tested once again (all *P* for approaching behavior between 0.001–0.027). We also found a significant effect of treatment on the closest distance to the speaker (Figure 3d; *P* = 0.009) and time spent within 10 m to the speaker (Figure 3e; *P* = 0.003). Birds came closer to the speaker when we played back the WC duets than the HC and the LC duets (Figure 3c–f). Tested pairs spent significantly more time within a 10-m radius, when responding to the WC duet, than to the HC and the LC duets (Table 1). In general, we did not observe significant differences in flight numbers between treatments (Table 2). However, we found a significant effect of the Treatment × Order interaction on flight numbers (*P* = 0.008). As with the case of solos, birds did many more flights (9.7 ± 2.58) when responding for the first time to the HC treatment (the general mean of flights per individual in all experiments was only 4.2 ± 0.51).

**Figure 4:**
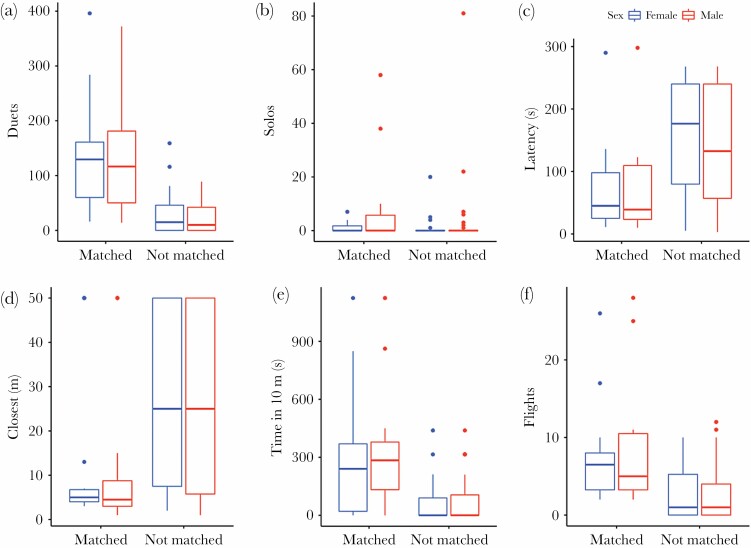
Female and male responses to playback in experiments during which focal males matched (*n* = 14) or did not match (*n* = 40) the song type of playback: (a) number of phrases produced in duets, (b) number of phrases produced solo, (c) latency to respond (s), (d) closest distance of approach to speaker (m), (e) time spent in a distance within 10 m to speaker (s), (f) number of flights to speakers. Box plots show the median with a horizontal line, the interquartile range (25th–75th percentile) with boxes, and the values within 1.5 times the interquartile range with whiskers. Dots show values exceeding 1.5 times the interquartile range.

We found that males matched the male part of duets provided in only 14 experiments, which means 25.9% of all experiments or 35.0% of experiments in which males sang any song in response. The latter value is thus very close and not significantly different from the predicted random pattern of matching (χ ^2^ = 0.07, df = 1, *P* = 0.92). In this study, males matched the HC playback two times, the LC playback three times, and the WC playback nine times, which gives 11.1%, 16.7%, and 50% of particular song type matching. Whichever way you look at this data (natural solos frequency perspective: Yates’ χ ^2^ = 26.39, df = 2, *P* < 0.001; duets perspective: Yates’ χ ^2^ = 41.89, df = 2, *P* < 0.001), it shows that males have avoided matching the most commonly used H type, as well as the L type, which, in turn, is comparably more often used in duets (though less in solos). On the other hand, they matched, more often than expected by chance, the WC playback. This result was also confirmed by the GLMM with matching behavior included as a binary response variable and treatment as an independent factor (*F* = 3.69, df = 2,51, *P* < 0.032). Moreover, pairs in which males matched the playback responded significantly stronger in all response variables. Such pairs produced more duets (Figure 4a) and solos (Figure 4b), as well as approached speakers faster, closer, and stayed close for a longer time (Figure 4c–f). Adding matching as an additional factor to GLMM models presented in Tables 1 and 2 makes it the strongest predictor of response strength (all *P* between 0.038 and <0.001) and in all cases cancels the significant effect of the treatment (all *P* ≥ 0.098).

## DISCUSSION

In this study, we experimentally tested the effect of male song type usage and song type matching during simulated territorial intrusion, for a duetting songbird, the yellow-breasted boubou. In general, we not only found that song types vary in terms of aggressive motivation but also that song type matching was a signal of a stronger threat during territorial response of focal pairs. The tested pairs did not always show a strong response but if they did, it was always linked with coordinated duetting and approaching. Interestingly, if a song type was matched, females reacted in a similar way with males, even though it was males choosing to match or not match the song of the intruder. Such a joint response has already been shown in experiments focused on testing differences in response to duets and male and female solos (Wheeldon et al. 2021). These results indicate a two-level way of coding aggression toward territory intruders and emphasize the importance of usage of the small and shared repertoire of male songs in territorial interactions.

### Differences in response to different song types

The advantage of the present study was that different types of duets were presented during simulated intrusions, and we found the strongest response to the WC duet playback, which was reflected by the number of duets, closest distance, and time spent close to speakers. Responses to the HC and the LC duets were weaker than to the WC duet but with some interesting differences between these two treatments too. Birds responded faster to the HC than the LC type, as well as staying at a larger distance from the speakers in the LC treatment. These results are very interesting when compared to what is already known about the amount of song type usage while naturally singing during the peak of the breeding season (Wheeldon et al. 2020) as well as in a year-round context (Szymański et al. 2021). The song type H is definitely the most commonly used by males when breeding (42.3% of all males’ song bouts), mostly as solos, as females only join males to form duets in 26.7% of H bouts (details in Wheeldon et al. 2020). We hypothesize that this male song type is primarily used to provide information about territory occupancy from a distance (stay away signal). The song type is proportionally less frequently joined by females to form duets and does not normally lead to between-pair conflict of conflict escalation. The song type L is generally less commonly used by males in solos but is more often (than H) answered by females to form a duet (64.6%; Wheeldon et al. 2020). Also the year-round monitoring of vocal activity revealed that the L song type is most often found in duets in comparison to the other two male song types (Szymański et al. 2021). Therefore, intrusion of a stranger pair with the LC duet (as this study’s data suggest) might be treated by territorial pairs in two ways: 1) as an incidental intrusion of a pair with members trying to communicate with each other or as 2) a well-coordinated, and thus dangerous, intrusion of pair intruders. The W song type was the least frequent song type used by males, and relatively rarely answered by females (25.9%) to form a duet. In addition, this song type was disproportionally often sung as the first song type during the dawn chorus (Wheeldon et al. 2020). In this study, we found that responses to the WC duets were the strongest and this was revealed by the number of duets, shortest latency, closest distance, and time spent close to the speakers. Coordination of duets requires attentiveness to the partner; therefore, it might be a signal both to the partner and to rivals within the signal range (Hall 2009). Our results show a slightly weaker response to the LC duets than to the HC duets, suggesting that there is no big difference in information contained in these two types of duets, in the context of territory defense. The most rarely produced song type W seems to be a signal of a higher threat (Wheeldon et al. 2020).

### Song type matching as a signal

A more in-depth analysis of response differentiation was possible if the song type matching pattern was involved. Matching was definitely not random and clearly demonstrated in experiments during which males matched the playback type where pairs responded stronger. The observed differences in strength of response to different duet types were in fact a result of how often focal individuals decided to match a particular song type, and if they did it, the response was strong. It is worth emphasizing that males were or were not matching the male part of the intruding duet, while both males and females were responding strongly together if the opponent signal was matched.

In our experiments, the response to the HC duet playback was in fact weak (e.g., number of duets 19.8 ± 5.39) if we do not include into the analysis the only two cases (number of duets 337.5 ± 27.43) when answering males matched the playback type. It seems that the HC duet type does not evoke a strong response as long as the responder does not match the song type of the intruder. This matching behavior was less frequent than expected by chance. When regarding the pattern of H usage we used for the calculation (random, solos, or duets) for the 18 HC playbacks, we should expect matching between 6 and 10 times, when in fact it was only matched 2 times. Similar to the HC treatment, the response to the LC duet type was only elevated (e.g., for duets 101.7 ± 44.02 vs. 27.0 ± 5.59) when focal males decided to match the playback and they matched the LC duet only in 3 out of the 18 experiments. The expected number of matching in 18 experiments should be between 5 and 7 for this type. Therefore, the LC duet only being matched three times indicates that it is not perceived as an extremely strong threat.

The question remains as to whether we observed the strongest response to the specific WC duet type or whether we observed the strongest response to this treatment because responding males matched this particular playback type most often (nine times)? The expected frequency of matching in the 18 WC treatments should be 6 (fully random pattern) or 2–3 (based on the natural frequency of solos and duets). When responding by matching the playback of the WC song type, birds approach very close to the speakers suggesting a readiness for physical attack. Simultaneously, the matching in the WC treatment was found in half the experiments suggesting a nonrandom pattern. Fast and close approach should be considered a greater threat because such direct proximity gives real possibility of attacking the opponent (Searcy and Beecher 2009). The results of the present study give a strong support for both hypotheses tested, indicating that for territorial defense in the yellow-breasted boubou the type of male song (H1) as well as the behavior of matching the song type of a male intruder (H2) is important.

We played back the three types of duets to each subject pair on different days but within a 24–48-h period to minimize variations that might occur due to changes in breeding status. We found significant order effects in all response variables except solos, which were produced quite sporadically. The order effect in such studies is not something unusual (e.g., Hall et al. 2006; Kovach et al. 2014). In our design, each playback type was presented in each order an equal number of times; hence, the order effect was minimized by means of interpretation of the all results. However, for future research, it is worth considering testing particular pairs only once.

### Consideration of communication networks

The results of this study together with earlier findings (Wheeldon et al. 2020, 2021; Szymański et al. 2021) allowed for the first interpretation of the communication network rules in the studied species. It is definite that the leading role, understood by the prevailing quantitative participation in all vocal bouts, belongs to males (Wheeldon et al. 2020, 2021; Szymański et al. 2021; this study). It also seems that loudly singing males signal to neighbors, strangers, and to their own mates, while singing females in most cases only tried to evoke responses from their own partners (Wheeldon et al. 2020). They rarely sing solos, both naturally and in experimentally induced provocations, in a way suggesting that they regularly signal to other females or nonmated males. Females simply stop singing quickly if not answered by their own mate (Wheeldon et al. 2020) and evoke weaker response of stranger pairs (Wheeldon et al. 2021). We do not want to suggest that female song does not play an important role in the study species. We have observed intense close interactions between pairs with intensive female singing or producing excitation calls (Wheeldon et al. 2020; own unpublished observations) were jamming the signal of the own partner by female might be a result of sexual conflict (Tobias and Seddon 2009). However, it seems that in a species living in a stable, year-round territorial system, such situations are simply rare and may be more frequent at other time. In fact, data from year-round recording indicates that female solo peak activity is at the end of breeding season (March–April; Szymański et al. 2021).

It seems that yellow-breasted boubou males have an efficient system of acoustical territory defense. With the use of a small but fully shared repertoire, they may inform others about territory occupancy as well as signal the willingness of a more aggressive response that may lead to a physical fight similar to other aggressive signaling systems (Wagner 1989; Waas 1991). Overall, these results show that the completely shared repertoire of male song types that contribute to duets is used as a territory defense signal by which the meaning of a signal (escalation or de-escalation of the conflict) results from both matching the intruder’s song as well as the specific song structure (or song type).

Interpretation of signal meaning is often problematic. One of the problems with aggressive signals is that signals produced by individuals in an aggressive context are not necessarily aggressive but, conversely, may signal submissiveness (Bradbury and Vehrencamp 2011) or even other aspects of behavior (Jakubowska and Osiejuk 2018). There are some useful frameworks in order to objectively tackle such problems. Searcy and Beecher (2009) proposed that the aggressive meaning of a signal must meet three criteria of the context, prediction, and response. According to them, an aggressive signal should be more frequent in an aggressive context, should predict escalation of the conflict by the sender, and should change the behavior of the targeted receivers. In this study, however, we were not able to test all three criteria, but it seems responding boubous meet the prediction criterion for song matching. Another useful framework was proposed by Vehrencamp et al. (2007). It is suggested that we may gain a more precise conclusion when considering signal meaning separately from the receiver’s and signaler’s perspective. In the present study, we directly tested the receivers’ perspective as we wanted to find out how territory owners respond to different signal types. We found that focal pairs responded with a differentiated strength to the different types of duet; however, this statement is only true if we consider the impact of song type matching. Testing the sender’s perspective is more difficult and the meaning of the signal to a signaler could be decoded by examining correlations between the signal characteristics and other aspects of the signaler’s behavior. In this study, we did not test this aspect experimentally, but we may infer about such information from the correlation between signal characteristics and other behaviors of the focal birds. Indeed, we found that responding birds that matched the playback song responded significantly stronger and were singing significantly more. In addition, in the case of matching, the duet type using the W male phrases approached speakers faster and closer. However, we are aware that further research demands an experimental approach allowing for direct testing of the signaler’s perspective. These results are also consistent with earlier findings for the banded wren (*Thryophilus pleurostictus*) indicating that song and approach behavior may show different response patterns depending on the threat level of the simulated intruder (de Kort et al. 2008). There is one important difference between the yellow-breasted boubou and the banded wren. It is suggested that the banded wren song’s deterrent value is constrained by a relationship between rate and bandwidth of trill notes (de Kort et al. 2008). In yellow-breasted boubous, all males are able to produce all different types of their shared repertoires; moreover, the rate of song phrases they produce is surprisingly invariable both within and between individuals (Wheeldon et al. 2020). In turn, this may indicate a completely different mechanism of song type signaling in both species. The described earlier in detail system of the yellow-breasted boubou has features indicating its conventional character, while the banded wren song is constrained by the production costs (de Kort et al. 2008; Ręk and Osiejuk 2010).

### Song matching in other duetting species

In the context of song type matching, repertoire functions have not been widely studied in duetting species. However, even sparse examples show that the extent to which individuals song type match their conspecifics songs varies across species and may not be related to repertoire size. For instance, although having a repertoire of 8–11 song types (Mennill and Vehrencamp 2005), relatively larger than that of the yellow-breasted boubou, both males and females of the neotropical rufous-and-white wren (*Thryophilus rufalbus*) have not been found to song type match the songs of same sex simulated intruders (Moser-Purdy et al. 2019). In the happy wren (*Pheugopedius felix*), a duetting species with a large repertoire, it has been shown that song type matching plays a role in within-pair communication rather than in signaling aggression to intruders, at least in the context (female response) tested (Templeton et al. 2013). On the other hand, plain wren pairs (*Thryothorus modestus zeldoni*) that have large and sex-specific song type repertoires used in duets use phrase type matching for strengthening their defense during territorial intrusions (Mann et al. 2003; Marshall-Ball and Slater 2004), this functionality of song type matching being similar to that of the yellow-breasted boubou. Conversely, both male and female eastern whipbirds (*Psophodes olivaceus*) match the same-sex songs, but females selected a song type matching the song of their partners, while males matched an intruder. Interestingly, relatively great variation in terms repertoire functions can also be found in closer relatives of yellow-breasted boubou, namely another *Laniarius* species in which the pattern of males singing whistles and females producing atonal notes seems to dominate (Winkler et al. 2020). For example, in the slate-colored boubou *L. funerbis*, different duet types have different functions, such as the synchronization of breeding, territorial defense, or mate guarding (Sonnenschein and Reyer 1983). Hence, links between song type and song functions in the slate-colored boubou seem to be much more straightforward than in the yellow-breasted boubous. In another closely related bush-shrike, the tropical boubou *L. aethiopicus*, repertoires are larger and much more complicated by means of male and female contribution, temporal precision, and functions, which include both territorial defense and mutual mate guarding (Grafe et al. 2004). The crimson-breasted shrike *L. atrocinneus* has sex-specific songs that are used for mate guarding behaviors (van den Heuvel et al. 2014). In conclusion, it seems that both the details of duet structure and their functions in *Laniarius* genus are varied and not based on a single pattern despite the superficial similarity.

### Sexual bias in response

We found no support inferring that the different male components of duets are directed to a different receiver, that is, a male or a female. All tested pairs responded with a very synchronized timing and strength, and even measuring how close and long birds stayed to the same-sex speaker did not reveal a sexual bias in response. It seems that if birds decided to respond, both pair members jointly defended their territory. The only obvious difference in response between sexes we found was that in a few experiments with the HC treatments, males produced more solos than females. However, this difference was not statistically significant in the full model for solos (*P* = 0.112; Table 2) and most of the vocal responses in these experiments was the result of duetting. This result confirms earlier findings showing that pairs respond with a similar strength to both duets and male solos, while responding significantly weaker to female solos (Wheeldon et al. 2021). This study gives further support that regardless of the duet type used by simulated intruders, males and females defend their territory together when faced with strangers. This obviously does not exclude the possibility that sometimes conflict of interest between mates may occur and that the less vocal females may force males to put in more effort, as was demonstrated by Tobias and Seddon (2009) and Wheeldon et al. (2021).

## CONCLUSIONS

We found that male and female yellow-breasted boubous responded more strongly in a vocal way to any type of duet if the male was matching the song type of the playback. Males most often matched the most rarely used song type W, whereas the other two song types were matched less often than expected by chance. Additionally, we found no significant sexual differentiation of response strength to any kind of territorial intrusion that was simulated. These results suggest that in yellow-breasted boubous, males use their small and shared repertoire in solos and duets in such a way that information is encoded in different song types as well as song type matching. As presented in the discussion, the research on song matching and the use of a repertoire for territorial defense in duetting species is very scarce. There is a great need for both basic descriptions of the use of song categories in the context of territory defense and for experiments testing precisely formulated hypotheses. It seems that even a small repertoire used by a duetting species is not a limitation for sophisticated territorial defense, as was also shown in birds without a song repertoire (Ręk and Osiejuk 2010). With this, we can see that these issues are still understudied in duetting animals and that a theoretically low variability of signals should not inhibit thorough research.

## Supplementary Material

arab030_suppl_Supplementary_Materials

## Notes

We thank Ernest Vunan Amohlon for his help in organizing field work in Cameroon and all the Kedjom-Keku Community for allowing to study birds on their land and Dr Moses Njoya from Bamenda University for help in organizing local permits. The video of duetting yellow-breasted boubous was done by Mathieu Mahamoud-Issa together with T.S.O.

Data Availability: Analyses reported in this article can be reproduced using the data provided by Osiejuk et al. (2021).
